# Myeloperoxidase Modulates Hydrogen Peroxide Mediated Cellular Damage in Murine Macrophages

**DOI:** 10.3390/antiox9121255

**Published:** 2020-12-10

**Authors:** Chaorui Guo, Inga Sileikaite, Michael J. Davies, Clare L. Hawkins

**Affiliations:** Department of Biomedical Sciences, University of Copenhagen, Panum, Blegdamsvej 3B, DK-2200 Copenhagen N, Denmark; chaorui@sund.ku.dk (C.G.); inga.sileikaite@sund.ku.dk (I.S.); davies@sund.ku.dk (M.J.D.)

**Keywords:** hypochlorous acid, hypothiocyanous acid, thiocyanate, glucose oxidase, inflammation, atherosclerosis, macrophage

## Abstract

Myeloperoxidase (MPO) is involved in the development of many chronic inflammatory diseases, in addition to its key role in innate immune defenses. This is attributed to the excessive production of hypochlorous acid (HOCl) by MPO at inflammatory sites, which causes tissue damage. This has sparked wide interest in the development of therapeutic approaches to prevent HOCl-induced cellular damage including supplementation with thiocyanate (SCN^−^) as an alternative substrate for MPO. In this study, we used an enzymatic system composed of glucose oxidase (GO), glucose, and MPO in the absence and presence of SCN^−^, to investigate the effects of generating a continuous flux of oxidants on macrophage cell function. Our studies show the generation of hydrogen peroxide (H_2_O_2_) by glucose and GO results in a dose- and time-dependent decrease in metabolic activity and cell viability, and the activation of stress-related signaling pathways. Interestingly, these damaging effects were attenuated by the addition of MPO to form HOCl. Supplementation with SCN^−^, which favors the formation of hypothiocyanous acid, could reverse this effect. Addition of MPO also resulted in upregulation of the antioxidant gene, NAD(P)H:quinone acceptor oxidoreductase 1. This study provides new insights into the role of MPO in the modulation of macrophage function, which may be relevant to inflammatory pathologies.

## 1. Introduction

There is compelling evidence showing the important role of the heme peroxidase enzyme myeloperoxidase (MPO), released by immune cells, in innate immunity and physiological processes associated with the development of chronic inflammatory diseases [1,2]. MPO is primarily released by neutrophils but is also present in monocytes and some tissue resident macrophages such as those associated with atherosclerotic lesions [3]. MPO catalyzes the reaction of halide (Cl^−^, Br^−^, I^−^) and pseudo-halide ions such as thiocyanate (SCN^−^) with hydrogen peroxide (H_2_O_2_) to produce the corresponding hypohalous acids [4]. Considering the physiological concentrations and specificity constants of these halides and pseudo-halides, it is believed that the main oxidants produced by MPO in vivo are hypochlorous acid (HOCl) and hypothiocyanous acid (HOSCN) generated from Cl^−^ and SCN^−^, respectively [5,6]. Supplementation with SCN^−^ favors the formation of HOSCN, and decreases the production of HOCl by MPO as the relative specificity constant for SCN^−^ is 730-fold greater than that for Cl^−^ [5]. In addition to acting as a competing substrate for MPO, SCN^−^ can react directly with HOCl, which also produces HOSCN [7].

HOCl and HOSCN show different reactivity with biological molecules (reviewed [8]). HOCl is a strong oxidant and potent antibacterial agent and plays an important role in innate immune processes [9]. However, it is also well established that HOCl can modify proteins, lipids, and DNA molecules irreversibly, which can contribute to host cell and tissue damage [2,4,10]. There is comprehensive literature regarding the reactivity of HOCl with different mammalian cell types, and little doubt that this oxidant causes cellular dysfunction and death by a multitude of cellular pathways (reviewed in [11]). It is therefore perhaps unsurprising that the production of HOCl by MPO at inflammatory sites is strongly linked to the development of many pathologies [1,2]. Unlike HOCl, HOSCN is a milder oxidant, which reacts selectively and reversibly with thiols (R-SH) including free Cys residues [12,13]. HOSCN can also kill pathogens but it is better tolerated by host cells, which has been postulated to be due to its selective detoxification by mammalian, but not bacterial, thioredoxin reductase (TrxR) [14].

This has led to interest in using SCN^−^ as a means to modulate MPO-induced damage, though HOSCN is also damaging to mammalian cells in some situations [15,16,17,18]. Nonetheless, previous studies have shown that supplementation with SCN^−^ in different cell types can be protective, and prevents MPO-induced cell damage [14,19,20]. Addition of SCN^−^ can also prevent extracellular MPO-induced damage including to plasma fibronectin [21] and extracellular matrix (ECM) proteins produced by human coronary artery smooth muscle cells [22]. Similarly, SCN^−^ exerts protective effects in some animal models of chronic inflammatory disease such as cystic fibrosis [23] and atherosclerosis [24,25], but not ulcerative colitis [26].

In general, the pathways responsible for the modulation of MPO-induced damage by SCN^−^, particularly in animal models, are not well characterized. Recent studies in murine J774A.1 macrophages indicated that SCN^−^ could attenuate thiol oxidation and cell death, and alter the nature of pro-inflammatory signaling induced by a bolus addition of HOCl, in a manner consistent with the formation of HOSCN [27]. This led us to examine whether these pathways are also relevant on the exposure of these macrophages to an enzymatic glucose/glucose oxidase (GO)–MPO coupled system, which provides a continuous flux of H_2_O_2_, and hence HOCl, and may be a better model to simulate the pro-inflammatory environment [14,20,28]. In the present study, we show an unexpected ability of MPO to attenuate the extent of cellular damage following exposure of the J774A.1 macrophage to a flux of H_2_O_2_ from the glucose/GO system. This modulatory effect of MPO was lessened in the presence of SCN^−^.

## 2. Materials and Methods

Reagents and Materials: All aqueous solutions were prepared using nano-pure H_2_O from a MilliQ system Millipore (Darmstadt, Germany). All chemicals and reagents were of the highest purity available and purchased from Sigma-Aldrich/Merck unless stated otherwise. Human polymorphonuclear leukocyte derived MPO was purchased from Planta Natural Products. Hanks buffered salt solution (HBSS) containing glucose (5.6 mM, “complete HBSS”) was from Thermo Fisher (Waltham, MA, USA). HBSS without glucose (“glucose-free HBSS”) was prepared by combining the following salts: NaCl (8 g), KCl (400 mg), CaCl_2_ (140 mg), MgSO_4_–7H_2_O (100 mg), MgCl_2_–6H_2_O (100 mg), NaHPO_4_–2H_2_O (60 mg), KH_2_PO_4_ (60 mg), and NaHCO_3_ (350 mg) in a final volume of 1 L.

Cell culture: Murine macrophage-like J774A.1 cells (ATCC No. 91051511) were cultured in Dulbecco’s modified Eagle’s medium (DMEM) supplemented with 10% (v/v) fetal bovine serum (FBS) Thermo Fisher, Waltham, MA, USA), 2 mM L-glutamine, and 100 U mL^−1^ penicillin (Invitrogen, Carlsbad, CA, USA) at 37 °C in a 5% CO_2_ incubator. For experiments, cells were scraped into suspension and adjusted to a density of 1 × 10^6^ cells mL^−1^ before seeding in 6-, 24-, or 96-well plates using volumes of 2000 µL, 500 µL, or 100 µL, respectively. Prior to treatment, cells were washed with complete HBSS at 37 °C. The same volumes of treatment solution containing glucose, GO, and MPO were used to ensure that the ratio of oxidant:cell remained constant in each case.

Cell viability: Cell viability was measured using the lactate dehydrogenase (LDH) assay and MTS (3-(4, 5-dimethylthiazol-2-yl)-5-(3-carboxymethoxyphenyl)-2-(4-sulfophenyl)-2H-tetrazolium) assay (Promega). J774A.1 cells were treated with GO (0–200 mU mL^−1^), with or without MPO (0–100 nM) in the absence and presence of SCN^−^ (200 µM) in complete HBSS (containing 5.6 mM glucose and 140 mM Cl^−^) for 1 h or 4 h.

For the MTS assay, cells (0.5 or 1 × 10^5^) in a 96-well plate were treated with the glucose/GO/MPO system for 1 h or 4 h in complete HBSS (total volume 100 µL) before washing and re-incubating in DMEM (100 µL) containing the MTS reagent (10 µL) for 4 h. The absorbance change was measured at 490 nm using a Spectra Max i3x microplate reader (Molecular Devices).

For the LDH assay, cells (5 × 10^5^) in 24 well plates were treated with the glucose/GO/MPO system for 1 h or 4 h in complete HBSS (total volume 500 µL) before washing and re-incubating in cell media (500 µL) for 24 h. The cell media were collected and centrifuged at 1000× *g* for 5 min in order to remove the cell debris. Cells were washed in complete HBSS and lysed in 500 µL water. The activity of LDH in cell lysates and media was determined by the loss of NADH measured at 340 nm over 30 min [29]. The cell integrity was calculated as the ratio of the change of absorbance at 340 nm for cell lysates to the total amount of cell lysates and media, as shown below:

(1) $$viability\%=\frac{\Delta 340\;\left(intracellular\right)}{\Delta 340\;\left(intracellular\right)+\Delta 340\;\left(extracellular\right)}\times 100$$

Quantification of cellular thiols: Cell lysates were prepared as described for the LDH assay and the intracellular thiol concentration was measured using the ThioGlo1 assay [30]. The cellular thiol concentration was assessed by the change in fluorescence measured using λ_ex_ 384 nm and λ_em_ 513 nm, as described previously [27]. Thiol concentrations were quantified using a standard curve constructed with GSH. Total protein concentrations were quantified by the BCA assay (Pierce™ BCA Protein Assay Reagent A, Thermo Fisher, Waltham, MA, USA) and were used to normalize thiol concentrations to adjust for any changes in cell number.

Measurement of H_2_O_2_ production using Amplex Red: The production of H_2_O_2_ by the glucose/GO system in the absence and presence of MPO was assessed by measuring the conversion of Amplex Red (10-acetyl-3,7-dihydroxyphenoxazine) to the fluorescent product resorufin, measured at λ_ex_ 571 nm and λ_em_ 585 nm. The fluorescence was measured following the addition of Amplex Red reagent (50 µL, 100 µM Amplex Red, and 0.2 U mL^−1^ HRP) to a reaction mixture containing GO (20, 50, and 100 mU mL^−1^) without and with MPO (50 nM) in complete HBSS (50 µL) after incubation for 30 and 60 min. Samples containing MPO were filtered through 10,000 Da molecular weight cut-off filters by centrifugation (10,000× *g* for 5 min at 4 °C) to remove MPO prior to the addition of Amplex Red. Control reactions were also performed using glucose-free HBSS. Reagent H_2_O_2_ (3%, 0.88 M stock) was used to prepare a standard curve.

Measurement of HOCl production by R19-S: The formation of HOCl by the glucose/GO/MPO system was assessed using the conversion of the pro-fluorescent probe R19-S to its fluorescent product, R19, measured at λ_ex_ 515 nm and λ_em_ 550 nm as described previously [31]. The fluorescence was measured at different time points (0–60 min) following the addition of R19-S (50 µL, 500 µM) to a reaction mixture containing MPO (50 nM), with or without GO (50 mU mL^−1^) and SCN^−^ (200 µM) in complete HBSS (final volume 50 µL). Reagent HOCl was used to prepare a standard curve, following standardization of the HOCl concentration by measuring its UV absorbance at 292 nm at pH 11, and using an extinction coefficient of 350 M^−1^ cm^−1^ [32].

Quantitative real-time polymerase chain reaction (qPCR): J774A.1 cells (5 × 10^5^) in 24 well plates were treated with 500 µL complete HBSS, or complete HBSS containing GO, GO/MPO, or GO/MPO/SCN^−^, as described above for the R19-S assay, for 1 h at 37 °C before washing with complete HBSS and re-incubating in DMEM for 24 h. Total RNA was extracted using the RNeasy Kit (Qiagen, Germantown, MD, USA), and genomic DNA was removed using RNAse Free DNase (Qiagen) before reverse transcription using a SensiFAST cDNA Synthesis Kit (Nordic Biosite, Täby, Sweden). Real-time PCR was performed on a 7900HT Fast Real-Time PCR System (Applied Biosystems) under the following thermal cycling conditions: 95 °C for 5 min, then 95 °C for 30 s, 60 °C for 30 s, and 72 °C for 30 s for 40 cycles, followed by 95 °C for 1 min and 55 °C for 1 min. A melt curve step consisting of stepwise temperature increases of 0.5 °C every 5 s beginning at 65 °C and ending at 95 °C was performed. The primer sequences are reported in Table S1. Relative mRNA concentrations of the genes of interest were normalized to 18S ribosomal RNA (18S), beta-2 microglobulin (B2M), and TATA-box binding protein (TBP) housekeeping genes. Data analysis was carried out using the 2^−ΔΔCT^ method.

Western blotting: For studies on the translocation of nuclear factor erythroid 2-related factor 2 (Nrf2) and c-JUN, J774A.1 cells (2 × 10^6^) in 6-well plates were treated as for the R19-S experiments but with the enzyme mixtures contained in a final volume of 2000 µL, for 1 h at 37 °C before washing with complete HBSS and re-incubation in DMEM for 24 h. Cells from two wells were pooled for analysis. Cytoplasmic and nucleic protein were extracted using commercial NE-PER™ Nuclear and Cytoplasmic Extraction Reagents (Thermo Fisher, Waltham, MA, USA) following the manufacturer’s protocol. Protein (10 µg) was separated by SDS-PAGE using NuPAGE 4–12% Bis-Tris gels (Thermo Fisher) at 200 V for 35 min, and transferred onto a polyvinylidene fluoride (PVDF) membrane (Thermo Fisher) at 20 V for 7 min. Membranes were blocked with 1% (*w/v*) bovine serum albumin (BSA) in TBST (0.1% Tween-20 in Tris-buffered saline) for 1 h at 21 °C, washed three times in TBST for 5 min, and then incubated with primary antibodies overnight at 4 °C: anti-Nrf2 (No. ab31163, Abcam, dilution 1:200), anti-c-JUN (No. 702170, Thermo Fisher, dilution 1:250), anti-TATA (No. ab818, Abcam, dilution 1:1000), and anti-β-actin (No. MAB8929, R&D, dilution 1:5000). The membranes were washed three times in TBST and incubated in HRP-conjugated anti-mouse (1:2000, No. NXA931, VWR) or anti-rabbit (1:2000, No. 7074S, BioNordika, Herlev, Denmark) IgG secondary antibodies for 1 h at 21 °C. The membrane was washed a further three times in TBST and imaged using SuperSignal™ West Pico PLUS Chemiluminescent Substrate (Thermo Fisher) using a Sapphire Biomolecular Imager (Azure Biosystems, Dublin, CA, USA). Band densities of cytoplasmic and nucleic protein were normalized to the loading control proteins β-actin and TATA, respectively.

Statistical analyses: Statistical analyses were performed using GraphPad Prism software 8, using 1-way and 2-way ANOVA tests with post-hoc analysis as detailed in the figure legends. Differences with *p* < 0.05 were taken as significant.

## 3. Results

### 3.1. Myeloperoxidase (MPO) Modulates the Extent of Glucose/GO-Induced J774A.1 Cell Death

**Figure 1:**
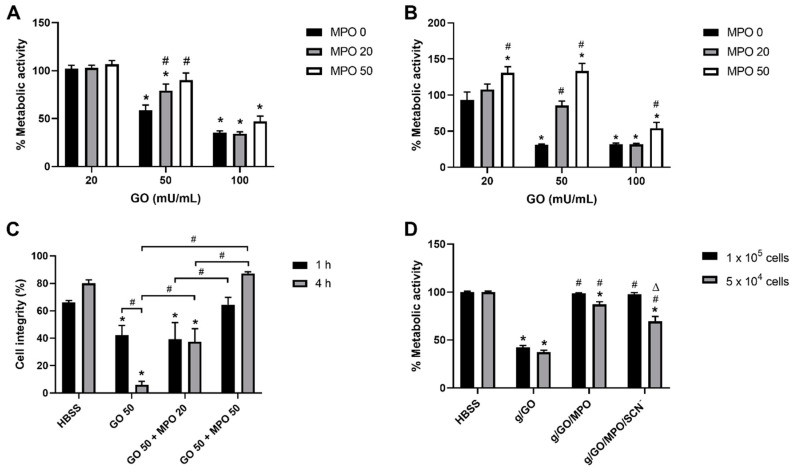
Myeloperoxidase (MPO) prevents glucose oxidase (GO)-induced macrophage cell death. (**A**,**B**) J774A.1 cells were treated with GO (20–100 mU mL^−1^) in complete HBSS, and different amounts of MPO (0, black bars; 20 nM, grey bars; 50 nM, white bars) for (**A**) 1 h or (**B**) 4 h before re-incubation in MTS-containing cell media for a further 4 h. (**C**) J774A.1 cells were treated with GO (50 mU mL^−1^) in the absence or presence of MPO (50 nM) in complete HBSS containing glucose for 1 h (black bars) or 4 h (grey bars) before re-incubation for 24 h and measuring LDH release. The cell integrity was determined by expressing the LDH released into the supernatant as a percentage of the total LDH. (**D**) J774A.1 cells (1 × 10^5^, black bars; 5 × 10^4^, grey bars) were incubated in complete HBSS with GO (50 mU mL^−1^), GO/MPO (50 nM), or GO/MPO/SCN^−^ (200 µM) for 1 h at 37 °C before re-incubation in cell media with MTS for 4 h. Data represent the mean ± S.E.M from three independent experiments. * shows a significant difference (*p* < 0.05) compared to the non-treated cells; # shows a significant difference (*p* < 0.05) compared to the glucose/GO groups (in A, B and D), or between the two groups indicated (in C); Δ shows a significant difference (*p* < 0.05) compared to the GO/MPO group, by a 2-way ANOVA with a Tukey’s multiple comparison test.

Initial studies were performed using the MTS assay to quantify changes in metabolic activity as a measure of cell viability, on exposure of the J774A.1 cells (1 × 10^5^) to a range of GO (0–200 mU mL^−1^) and MPO (0–100 nM) concentrations and treatment times, in the presence of complete HBSS, which contained glucose (5.6 mM) and Cl^−^ (140 mM). Treatment of the J774A.1 cells with the glucose/GO system in the absence of MPO induced a loss of metabolic activity in a dose- and time-dependent manner, which was significant in experiments with ≥50 GO mU mL^−1^ and 1 h or 4 h incubation (Figure 1A,B, Figure S1). The extent of loss in metabolic activity was greater after 4 h incubation, with a significant change compared to non-treated cells seen with 20 mU mL^−1^ GO (Figure 1B, Figure S1). No change in metabolic activity was seen in cells exposed to GO in HBSS without glucose for 1 h or 4 h, indicating that the toxicity was associated with H_2_O_2_ generation (Figure S1). Similarly, there was no change in metabolic activity in experiments with MPO in the absence of GO, presumably due to the absence of significant concentrations of H_2_O_2_ formation, the substrate for MPO (Figure S1).

The addition of MPO to the cells in the presence of the glucose/GO system to promote the formation of HOCl resulted in either no change (with 20 or 200 mU mL^−1^ GO) or an increase (with 50 mU mL^−1^ GO) in the metabolic activity compared to that seen in the cells treated only with glucose/GO (Figure 1A,B, Figure S1). The ability of MPO to modulate the GO-induced loss in metabolic activity was also dependent on the MPO concentration (Figure 1). No evidence was obtained for a greater loss in metabolic activity in the J774A.1 cells on the addition of MPO to the enzymatic GO system, above that seen with GO alone, under any of the conditions examined, in contrast to previous studies [14,20].

To confirm that the changes in metabolic activities were related to viability, LDH assays were performed to assess cell lysis. The J774A.1 cells were exposed to the glucose/GO system (50 mU mL^−1^ GO) with and without the addition of MPO (20 and 50 nM) for 1 h or 4 h. In this case, the cells were washed to remove the enzymes, and re-incubated for 24 h in complete cell media, before LDH release was measured. The formation of H_2_O_2_ by GO resulted in a significant decrease in cell integrity, with a much higher extent of LDH release, consistent with increased levels of cell lysis after 4 h treatment compared to 1 h (Figure 1C). This cell lysis was attenuated by the addition of MPO in a dose- and time-dependent manner with the cell integrity returning to the levels observed in the non-treated, control cells in experiments with 50 nM MPO (Figure 1C).

### 3.2. Effect of Supplementation with SCN^−^ on Glucose/GO/MPO-Induced Cell Death.

Given the previous data showing that SCN^−^ is able to modulate the extent of MPO-induced cellular damage [14,19,20], these studies were extended to examine the ability of SCN^−^ to influence the alteration of metabolic activity seen in the J774A.1 cells on exposure to the glucose/GO/MPO system. In these experiments, the effect of altering the ratio of oxidant to cell was also examined by comparing the metabolic activity from experiments with different cell numbers (1 × 10^5^ and 5 × 10^4^ cells). A significant loss in metabolic activity was observed on exposure of the J774A.1 cells to the glucose/GO system after 1 h, which was comparable in experiments with both cell densities (Figure 1D). The loss in metabolic activity was attenuated by the presence of MPO, though in this case, the effect was dependent on the density of cells used, and did not return completely to the levels seen in the control cells in experiments with a lower cell density (Figure 1D). The addition of SCN^−^ to the J774A.1 cells had no effect on the ability of MPO to modulate the change in metabolic activity in the cells exposed to glucose/GO at the higher cell density (1 × 10^5^ cells). However, there was a significant decrease in the modulatory effect of MPO in the presence of SCN^−^ in the experiments with a lower cell number (5 × 10^4^), where the ratio of oxidant:cell was greater (Figure 1D).

### 3.3. Production of H_2_O_2_ and HOCl by the Glucose/GO/MPO System

**Figure 2:**
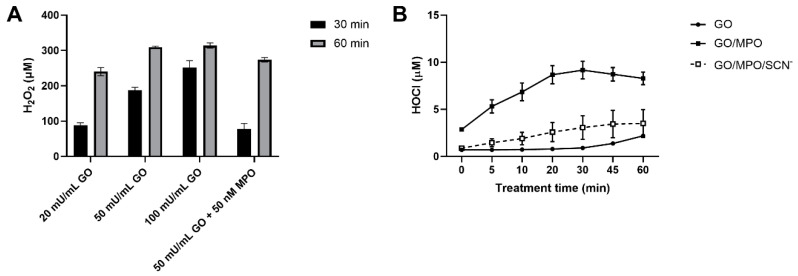
The production of H_2_O_2_ and HOCl by the GO/MPO enzymatic system. (**A**) The formation of H_2_O_2_ was assessed using the Amplex Red reagent (100 µM Amplex Red and 0.2 U mL^−1^ HRP) in reaction mixtures containing GO (20, 50, and 100 mU mL^−1^) in complete HBSS after 30 and 60 min by measuring resourfin fluorescence at λ_ex_ 571 nm and λ_em_ 585 nm. Samples containing GO (50 mU mL^−1^) and MPO (50 nM) were also examined. (**B**) HOCl was assessed using the R19-S probe (250 µM) in reaction mixtures containing GO (50 mU mL^−1^) and MPO (50 nM) in the absence and presence of SCN^−^ (200 µM) in HBSS containing glucose (5.6 mM). Fluorescence was measured using λ_ex_ 515 nm and λ_em_ 550 nm at different time points (0–60 min), with GO (circle, solid line), GO/MPO (solid symbol, solid line), and GO/MPO/SCN^−^ (open square, dashed line). Data represent mean ± S.E.M from three independent experiments.

To further examine the cellular effects of the glucose/GO/MPO system, the production of H_2_O_2_ was quantified using Amplex Red. A dose- and time-dependent increase in the production of H_2_O_2_ was observed on the addition of GO (20, 50, and 100 mU mL^−1^) to complete HBSS containing glucose (Figure 2A). The production of H_2_O_2_ was dependent on the presence of glucose in the HBSS, and decreased on the addition of MPO (50 nM; Figure 2A), consistent with the conversion of H_2_O_2_ to HOCl. Care was taken to remove the MPO prior to quantification of H_2_O_2_ to avoid the peroxidase reaction of MPO with the Amplex Red substrate. The formation of HOCl was assessed using the fluorescent probe R19-S (Figure 2B). The non-fluorescent, R19-S probe reacts with HOCl to give a fluorescent product, R19, which can be quantified [31]. A time-dependent increase in the R19 fluorescence was observed on the addition of MPO (50 mM) to a reaction mixture containing GO (50 mU mL^−1^) in complete HBSS, consistent with the formation of a HOCl (10 µM; Figure 2B). In contrast, there was minimal increase in fluorescence in the experiments with glucose/GO in the absence of MPO. Inclusion of SCN^−^ (200 µM) into the complete glucose/GO/MPO system resulted in a significant decrease in the rate and extent of fluorescence when compared to the system without this anion (Figure 2B). This can be attributed to a decrease in HOCl production and the formation of HOSCN, which is poorly reactive with R19-S [31].

### 3.4. Pathways Involved in Alteration of H_2_O_2_-Induced Cell Damage by MPO.

**Figure 3:**
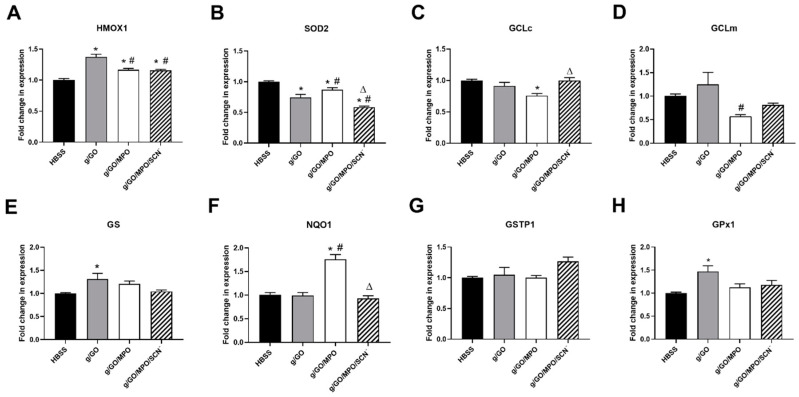
The effect of the GO/MPO enzymatic systems on antioxidant gene expression. J774A.1 cells (5 × 10^5^) were incubated in HBSS containing glucose (5.6 mM) (black bars), with GO (50 mU mL^−1^, grey bars), GO/MPO (50 nM, white bars), or GO/MPO/SCN^−^ (200 µM, hatched bars) for 1 h at 37 °C before re-incubation in cell media for 24 h. Expression of antioxidant genes, HMOX1 (**A**), SOD2 (**B**), GCLc (**C**), GCLm (**D**), GS (**E**), NQO1 (**F**), GSTP1 (**G**), and GPx1 (**H**) was measured using qPCR. Data are expressed as the fold change compared to the non-treated group following normalization to the housekeeping genes TBP, 18s, and B2M and are represented as the mean ± SEM from three independent experiments. * shows a significant difference (*p* < 0.05) compared to the non-treated (HBSS) group; # shows a significant difference (*p* < 0.05) compared to cells treated with the GO system in the absence of MPO; Δ shows a significant difference (*p* < 0.05) compared to cells treated with the GO/MPO systems by 1-way ANOVA and Tukey’s multiple comparison test.

The ability of MPO and SCN^−^ to modulate cellular damage induced by H_2_O_2_ produced by glucose/GO was examined further by using qPCR to assess changes in the expression of genes associated with stress and antioxidant responses including genes associated with activation of Nrf2. J774A.1 cells were exposed to either GO (50 mU mL^−1^), GO with MPO (50 nM), or GO/MPO with SCN^−^ (200 µM) in complete HBSS. Evidence was obtained for a significant increase in the expression of heme oxygenase 1 (HMOX1) in experiments with GO, which was significantly reduced in the presence of MPO, regardless of whether SCN^−^ was present, but still remained elevated compared to the non-treated cells (Figure 3A). An increase in the mRNA expression of glutathione synthetase (GS) and glutathione peroxidase 1 (GPx1) was also observed on the exposure of the cells to the GO system, which was attenuated by the presence of MPO (and SCN^−^) (Figure 3E,H).

In contrast, treatment of the J774A.1 cells with the glucose/GO system in the absence of MPO resulted in a significant decrease in the expression of SOD2; this was partly attenuated in the presence of MPO, but decreased further on the addition of MPO and SCN^−^ (Figure 3B). There was no change in the expression of other antioxidant genes including the glutamate–cysteine ligase catalytic subunit (GCLc), glutamate–cysteine ligase modifier subunit (GCLm), glutathione S–transferase Pi 1 (GSTP1) or NAD(P)H: quinone oxidoreductase (NQO1) on exposure of the cells to the GO system. However, the addition of MPO resulted in a significant elevation in the expression of NQO1, which was attenuated by the presence of SCN^−^, suggesting that the production of HOCl is responsible for the alteration in the expression of this gene (Figure 3F). The addition of MPO also resulted in a significant decrease in the expression of GCLc (Figure 3C) and GCLm (Figure 3D) compared to the HBSS control or glucose/GO treated cells, respectively.

**Figure 4:**
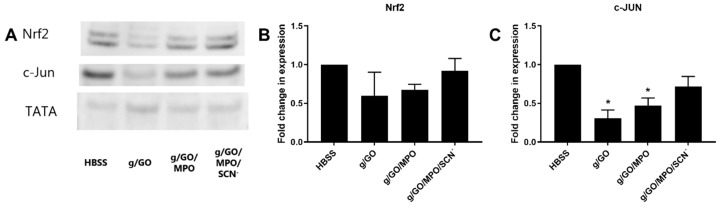
Exposure of J774A.1 cells to the GO/MPO enzymatic system suppressed the translocation of c-JUN. J774A.1 cells (2 × 10^6^) were incubated in HBSS containing glucose (5.6 mM) (black bars), with GO (50 mU mL^−1^), GO/MPO (50 nM), or GO/MPO/SCN^−^ (200 µM) for 1 h at 37 °C before re-incubation in cell media for 24 h. The nuclear protein was extracted using a commercial kit and 10 µg protein was loaded. TATA was used as a loading control. Images are representative of three independent experiments (**A**). Panels B and C show the densitometry analysis of Nrf2 (**B**) and c-JUN (**C**) following normalization to TATA. Data are expressed as the fold change compared to the respective non-treated group. * shows a significant difference (*p* < 0.05) compared to the non-treated control groups by 1-way ANOVA with a Dunnett’s multiple comparison test.

In light of the evidence for some alteration in the expression of genes associated with GSH biosynthesis, these studies were extended to examine the effects of the enzyme treatments on intracellular thiol levels using ThioGlo 1 under analogous experimental conditions. No significant change in the levels of intracellular thiols were observed on treating the J774A.1 cells with GO (50 mU mL^−1^) in the absence or presence of MPO (20 and 50 nM) for 1 h in complete HBSS, followed by 24 h re-incubation in cell media (Figure S2). Additional experiments were also performed to examine the induction and nuclear translocation of Nrf2 and c-JUN, which are known to regulate the expression of antioxidant genes [33,34]. The J774A.1 cells were exposed to the GO, GO/MPO, and GO/MPO/SCN^−^ systems using the conditions described above for the qPCR experiments, before assessing the translocation of Nrf2 and c-JUN from the cytoplasm to nuclei by western blotting. There was a trend toward a decrease in Nrf2 nuclear translocation with the GO and GO/MPO systems compared to the non-treated control cells, but these changes were not significant (Figure 4). With c-JUN, a significant decrease in nuclear translocation was seen on treating the cells with GO and GO/MPO, which was increased slightly by the addition of SCN^−^ (Figure 4). No changes were observed in the cytoplasmic levels of Nrf2 or c-JUN on comparing each enzymatic system to the non-treated, HBSS control (Figure S3).

## 4. Discussion

There is significant interest in the development of new therapeutic strategies to modulate the production of HOCl from MPO in light of the key role of this oxidant in the development of numerous chronic inflammatory pathologies [2]. Therefore, in this study, we employed an enzymatic glucose/GO system in combination with MPO to examine the effects of a continuous flux of H_2_O_2_ compared to HOCl on macrophage cell function, and establish how this is perturbed by supplementation with SCN^−^. However, our data are consistent with a modulatory, rather than damaging, role for MPO. Thus, the generation of HOCl by MPO was associated with a decreased extent of macrophage damage compared to that induced by the H_2_O_2_ produced by glucose/GO. Moreover, decreasing the production of HOCl by the addition of SCN^−^ to favor HOSCN formation could influence this effect of MPO. These data are in contrast with previous studies with GO and MPO, where MPO increased cell death, and SCN^−^ had a clear protective role [14,20].

The ability of MPO to propagate or exacerbate the development of chronic inflammatory disease is mainly attributed to the excessive formation of HOCl, which is cytotoxic and highly damaging to both bacterial and mammalian cells [1,2,4]. A large number of studies have provided evidence for the disruption of key cellular functions and signaling pathways on the exposure of many different mammalian cell types to reagent HOCl, added as a bolus to cells in balanced salt solutions or cell media (reviewed in [11]). However, to better mimic the oxidative burst at inflammatory sites, it is also relevant to consider the cellular effects of a flux of HOCl (and other oxidants) generated enzymatically. To this end, experimental models have been developed that utilize the glucose/GO/MPO system [14,20,22,28]. In this system, physiologically relevant concentrations of glucose and Cl^−^ are provided by performing studies in a balanced salt solution such as complete HBSS. This enables the continuous generation of H_2_O_2_ from GO, which is converted to HOCl in the presence of MPO and/or HOSCN when SCN^−^ is also present [14,20].

Exposure of J774A.1 macrophages to the glucose/GO system resulted in a dose- and time-dependent loss in cell viability, as assessed by measuring metabolic activity with the MTS assay or determining the extent of leakage of LDH, 24 h post-treatment. The loss in viability seen with GO was dependent on the presence of glucose, supporting a damaging role for H_2_O_2_ production. The J774A.1 cells were exposed to between 200–300 µM H_2_O_2_, depending on the concentration of GO used. The concentration of H_2_O_2_ formed over 1 h with 50 mU mL^−1^ GO compared well with the theoretical flux of 5 µM min^−1^ H_2_O_2_ calculated on the basis of the enzymatic units of GO used. The rate of generation of H_2_O_2_ was more rapid with higher concentrations of GO (100 mU mL^−1^), but the concentration of the oxidant appeared to reach a plateau at ca. 300 µM on quantification at 1 h. A comparable loss in cell viability has been reported previously in J774A.1 cells exposed to the same concentration of GO [35]. It could be helpful in future studies to assess the effects of the addition of catalase, rather than MPO, as a means to modulate the toxicity of glucose/GO.

The addition of MPO did not enhance, but rather, decreased, the extent of GO-induced cell death in experiments with a range of GO and MPO concentrations. This effect of MPO was partially reversed on supplementation of the cells with SCN^−^ (200 µM), which decreased HOCl production in favor of the generation of HOSCN. In this study, GO, MPO, and SCN^−^ (if applicable) were added to cells together in complete HBSS (containing glucose and Cl^−^). It is therefore likely that oxidant generation (H_2_O_2_, HOCl, and/or HOSCN) will primarily occur externally to the cell under these conditions. This may have a significant impact on the resulting cell effects as the lower reactivity of H_2_O_2_ and HOSCN, when compared to HOCl, is likely to result in these being able to penetrate into the cells (via passive diffusion) and exert cellular effects. The relative uptake of H_2_O_2_ compared to HOSCN has not been examined, but is likely to be slower for HOSCN as the pK_a_ is 5.3, which would favor the presence of hypothiocyanite (^−^OSCN) at pH 7.4 [36]. In contrast, the high reactivity (and hence very limited diffusion radius) of HOCl, is likely to result in very limited or no cellular penetration, with the reaction occurring primarily in the extracellular milieu or at the cell surface. The modulatory effect of MPO on the effects of the glucose/GO system could reflect a change from internal (with H_2_O_2_ and HOSCN) oxidative events to extracellular reactions with HOCl, and hence altered cellular susceptibility to oxidative damage.

These data are in contrast with previous studies using this enzymatic model with other cell types where MPO increased cell death, and a protective effect of SCN^−^ was observed [14,20]. Of note, a higher concentration of MPO was employed (1 U mL^−1^ [20] and 5 U mL^−1^ [14]) compared to that used in the current study (typically 50 nM, equivalent to ca. 9 mU mL^−1^ MPO), which may result in exposure of the cells to a greater concentration of HOCl. Thus, although the production of HOCl will be dependent on the flux of H_2_O_2_, it is possible that a higher concentration of MPO will result in less inactivation of the MPO by HOCl. This may be relevant in our studies as the amount of HOCl detected (10 µM) is lower than the H_2_O_2_ consumed (110 µM) on the addition of MPO, where the HOCl concentration reaches a plateau (after 30 min). This may contribute, at least in part, to the differences observed, given that the dose of oxidant per cell is known to influence the extent of cell death (reviewed in [11]). In addition, there are likely to be differences in the site of HOCl generation, resulting from alterations in MPO localization. For example, in the previous experiments with human lung epithelial Calu-3 cells, mouse neuroblastoma Neuro2a cells, mouse pancreatic β Min6 cells, and human aortic endothelial cells, the cells were incubated with MPO for 1 h prior to the addition of GO to initiate the formation of H_2_O_2_ [20]. MPO readily binds to the surface of many different cells, which can affect cellular function independently of its catalytic activity (e.g., [37,38,39]). With red blood cells, binding was prevented by pre-treatment of MPO with HOCl due to a decrease in the positive charge on MPO arising from the modification of Lys residues [37]. This suggests that MPO binding might be disfavored on co-treatment of the cells together with GO/MPO. Moreover, MPO binding to the cell surface can also facilitate its internalization by cells. Thus, MPO binding to the mannose-6-phosphate receptor has been reported to result in subsequent lysosomal accumulation [40]. Other studies have provided evidence for both cytoplasmic and nuclear accumulation of MPO [39]. In each case, the MPO retains its catalytic activity, which is likely to favor intracellular HOCl formation, although some cellular effects appear to be unrelated to oxidant generation [39,40].

A differential ability of cells to detoxify oxidants, particularly H_2_O_2_ and HOSCN, which are less reactive than HOCl [2,4], may also contribute to the differences in the data reported here, compared to previous studies with other cell types. Thus, in studies where human bronchial epithelial 16HBE cells were treated with glucose/GO/MPO, the cells were cultured with media containing selenium methylselenocysteine (100 nM), which promotes the synthesis and activity of antioxidant selenoprotein enzymes including TrxR [14]. TrxR utilizes NADPH to convert oxidized thioredoxin to reduced thioredoxin, and as such, plays an integral role in oxidant detoxification and redox regulation in cells (reviewed in [41]). TrxR is an important mechanism by which mammalian cells detoxify HOSCN to decrease cytotoxicity and is postulated to account for the cellular protection seen on the addition of SCN^−^ to the glucose/GO/MPO system [14]. In our study, the cells were not cultured with any selenium-containing compound to elevate selenoprotein synthesis. In addition, the J774A.1 cell line is reported to have a decreased TrxR activity compared to that seen in other cell lines [23]. This could result in J774A.1 cells having a greater sensitivity to H_2_O_2_ and HOSCN compared to other cell types.

The modulatory effect of MPO against glucose/GO-mediated J774A.1 macrophage damage may reflect an adaptive response in the cells triggered by exposure to a low concentration of HOCl, rather than simply by decreasing the concentration of H_2_O_2_. This is supported by the observation that the concentration of H_2_O_2_ produced in the presence of MPO is only ~40 µM lower than that seen with the glucose/GO system alone after 1 h incubation in a cell-free system (Figure 2A). Moreover, supplementation with SCN^−^, which significantly decreased HOCl formation, could decrease this modulatory effect under some experimental conditions (Figure 1D). SCN^−^ decreases HOCl by acting as a competitive substrate for MPO and by directly scavenging HOCl [7]. Recent studies with cumulus oocytes demonstrated a similar ability of MPO (40 nM) to prevent H_2_O_2_-induced cell death [42]. In this case, the modulatory role of MPO was observed in experiments where the oocytes were exposed to low concentrations (10 µM) of HOCl, which resulted in the inactivation of caspase 3 in the absence of cell death [42]. The HOCl-dependent decrease in caspase 3 activity resulted in a decrease in the extent of apoptosis seen on exposure to H_2_O_2_ alone [42].

To better understand the mechanisms responsible for the protective role of MPO against glucose/GO-mediated cellular damage, we examined antioxidant gene expression and signaling pathways. Exposure of the J774A.1 cells to glucose/GO in the absence or presence of MPO resulted in some differences in the mRNA expression of individual antioxidant genes including HMOX1, SOD2, GCLc, GCLm, GS, NQO1, and GPx1, which in some cases, was also affected by SCN^−^. However, overall, the pattern of antioxidant gene expression observed under different treatment conditions was not consistent with alterations in Nrf2 activation. Treating the cells with glucose/GO/MPO resulted in a significant increase in the expression of NQO1, which was not seen in experiments with glucose/GO alone, or when SCN^−^ was added. This is attributed to the exposure of the cells to a low, sub-lethal, flux (10 µM) of HOCl and was not observed in experiments with J774A.1 cells treated with higher concentrations (50 µM) of reagent HOCl [27]. It will be important in future studies to examine alterations in gene expression in experiments with sub-lethal bolus concentrations HOCl (<20 µM).

It is not clear whether the elevation in NQO1 gene expression is responsible for the modulatory role of MPO on glucose/GO-induced cell lysis and loss of metabolic activity. NQO1 can provide protection against oxidative stress through various pathways, in addition to its role in quinone reduction [43]. NQO1 can reduce superoxide radicals, which may be important when SOD levels are low. However, the rate constant for this reaction is slow, and this pathway is unlikely to be relevant here as no superoxide radical generating system was used [44]. NQO1 is also involved in the stabilization of proteins with functional and transcriptional effects and can affect protein translation by binding to mRNA [44]. This is suggested to have a protective function and could potentially promote cellular survival. However, the pathway responsible for the upregulation of NQO1 mRNA expression in this study has not been established. NQO1 is highly inducible by multiple cellular stressors and can be upregulated independently of Nrf2 activation [43,44]. An upregulation of NQO1 has been observed previously in murine RAW264.7 macrophages on exposure of the cells to low concentrations of HOCl, though in this case, the change in NQO1 expression was accompanied by similar increases in the mRNA of Nrf2 regulated genes including GCLm and HMOX1 [45]. Further work needs to be done to assess whether the changes in NQO1 mRNA expression are associated with an upregulation of the protein and altered enzymatic activity.

## 5. Conclusions

In summary, MPO was able to attenuate the cell death induced by the enzymatic formation of H_2_O_2_ by glucose/GO, which may be related to the ability of HOCl to upregulate the expression of antioxidant genes like NQO1 in a manner independent of Nrf2 and c-JUN activation. Supplementation with SCN^−^ could reverse this modulatory effect, which appears to be associated with a decrease in HOCl production and elevation in HOSCN formation, though the latter was not quantified. This study provides evidence in support of a potential modulatory role for MPO on the extent of cellular damage attributed to the production of low amounts of HOCl. It also highlights the need for additional mechanistic studies to better understand the utility of SCN^−^ as a therapeutic strategy to target MPO-induced cellular damage in chronic inflammatory disease.

## Supplementary Materials

The following are available online at https://www.mdpi.com/2076-3921/9/12/1255/s1, Table S1: Mus primer sequences used for qPCR; Figure S1: Effect of the glucose/GO/MPO enzymatic system on J774A.1 metabolic activity; Figure S2: Effect of the glucose/GO/MPO enzymatic system on intracellular thiols in J774A.1 cells; Figure S3. Cytoplasmic Nrf2 and c-JUN are not altered in J774A.1 cells on exposure to the glucose/GO/MPO enzymatic system.

## Abbreviations

18S, 18S ribosomal RNA; B2M, beta-2-microglobulin; BSA, bovine serum albumin; DMEM, Dulbecco’s modified Eagle’s medium; GCLc, glutamate–cysteine ligase catalytic subunit; GCLm, glutamate–cysteine ligase modifier subunit; GO, glucose oxidase; GPx1, glutathione peroxidase 1; GS, glutathione synthetase; GSTP1, glutathione S–transferase Pi 1; HBSS, Hanks balanced salt solution; HMOX1, heme oxygenase 1; HOCl, hypochlorous acid; HOSCN, hypothiocyanous acid; LDH, lactate dehydrogenase; MPO, myeloperoxidase; MTS, 3-(4,5-dimethylthiazol-2-yl)-5-(3-carboxymethoxyphenyl)-2-(4-sulfophenyl)-2H-tetrazolium); NQO1, NAD(P)H quinone oxidoreductase; Nrf2, erythroid 2-related factor; SCN^−^, thiocyanate; SOD2, mitochondrial superoxide dismutase 2; TBP, TATA-box binding protein; TrxR, thioredoxin reductase.
