# Supplementary material for: Myeloperoxidase Modulates Hydrogen Peroxide Mediated Cellular Damage in Murine Macrophages
Source: Antioxidants (Basel). 2020 Dec 10;9(12):1255. doi: 10.3390/antiox9121255 (PMC7764223; doi:10.3390/antiox9121255)
Supplement: Supplementary file 1 [file antioxidants-09-01255-s001.pdf]

SUPPLEMENTARY DATA

Myeloperoxidase modulates hydrogen peroxide mediated cellular damage in murine macrophages.

Chaorui Guo, Inga Sileikaite, Michael J. Davies and Clare L. Hawkins*

Department of Biomedical Sciences, University of Copenhagen, Panum, Blegdamsvej 3B, Copenhagen N, DK-2200, Denmark

Keywords: Hypochlorous acid; thiocyanate; glucose oxidase; inflammation; atherosclerosis; macrophage

* To whom correspondence should be addressed: Prof. Clare Hawkins, Department of Biomedical Sciences, University of Copenhagen, Panum, Blegdamsvej 3B, Copenhagen N, DK-2200, Denmark.

Email: clare.hawkins@sund.ku.dk

Table S1. Mus primer sequences used for qPCR.

Gene	Forward sequence (5'-3')	Reverse sequence (5'-3')
TATA-box binding protein (TBP)	CACAGGAGCCAAGAGTGAAGA	CACAAGGCCTTCCAGCCTTA
18S ribosomal RNA (18S)	GTAACCCGTTGAACCCCATT	CCATCCAATCGGTAGTAGCG
Mus beta-2 microglobulin (B2M)	TGGTCTTTCTGGTGCTTGTC	GGATTTCAATGTGAGGCGGGT
Heme oxygenase 1 (HMOX1)	ACAGCCCCACCAAGTTCAAA	TCTGCAGGGGCAGTATCTTG
Superoxide dismutase 2, mitochondrial (SOD2)	ACAACTCAGGTCGCTCCTCAG	GATAGCCTCCAGCAACTCTCC
Glutathione Peroxidase 1 (GPx1)	CCACCGTGTATGCCTTCTCC	AGAGAGACGCGACATTCTCAAT
Glutathione S-Transferase Pi 1 (GSTP1)	ATGCCACCATACACCATTGTC	GGGAGCTGCCCATACAGAC
Glutamate-Cysteine Ligase Catalytic Subunit (GCLc)	GGACAAACCCCAACCATCC	GTTGAACTCAGACATCGTTCCT
Glutamate-Cysteine Ligase Modifier Subunit (GCLm)	CTTCGCCTCCGATTGAAGATG	AAAGGCAGTCAAATCTGGTGG
NAD(P)H Quinone Dehydrogenase 1 (NQO1)	AGGATGGGAGGTACTCGAATC	TGCTAGAGATGACTCGGAAGG
Glutathione synthetase (GS)	GGTATCTTCCCTCAGCAGCCTT	GCTTCCATTCCCACACTCCAAA

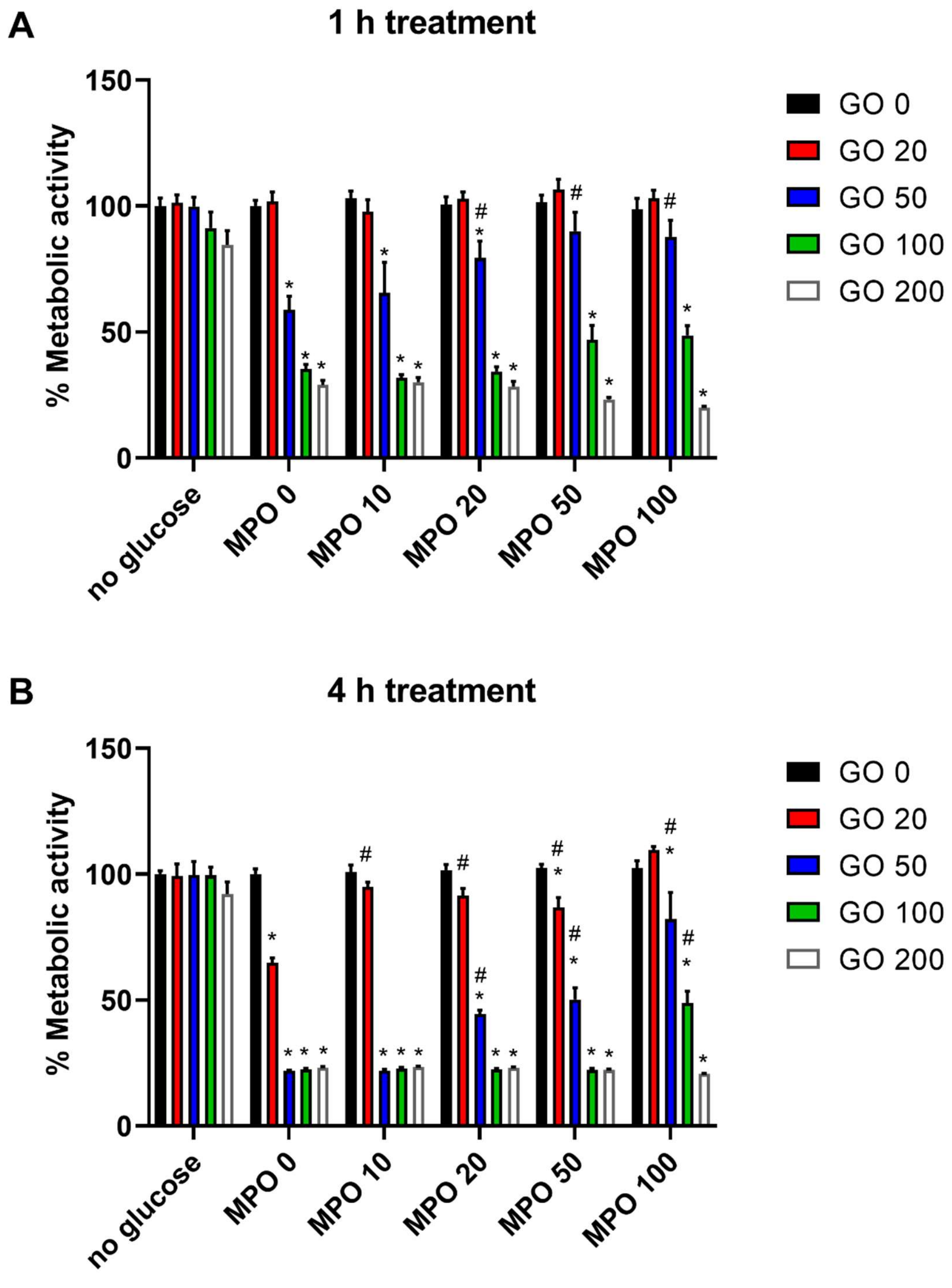

Figure S1: Effect of the glucose/GO/MPO enzymatic system on J774A.1 metabolic activity.

J774A.1 cells (1×10^5) were treated with GO (0-200 mU mL^{-1}) in HBSS with or without glucose (5.6 mM), in the absence and presence of MPO (0-100 nM) for either (a) 1 h or (b) 4 h before re-incubation in MTS containing cell media for 4 h. Data are expressed as the percentage of metabolic activity compared to the non-treated group and represent the mean ± S.E.M from 3 independent experiments. * shows a significant difference ($p < 0.05$) compared to the non-treated cells; # shows a significant difference ($p < 0.05$) compared to the glucose/GO group without MPO, by a 2-way ANOVA with a Tukey's multiple comparison test.

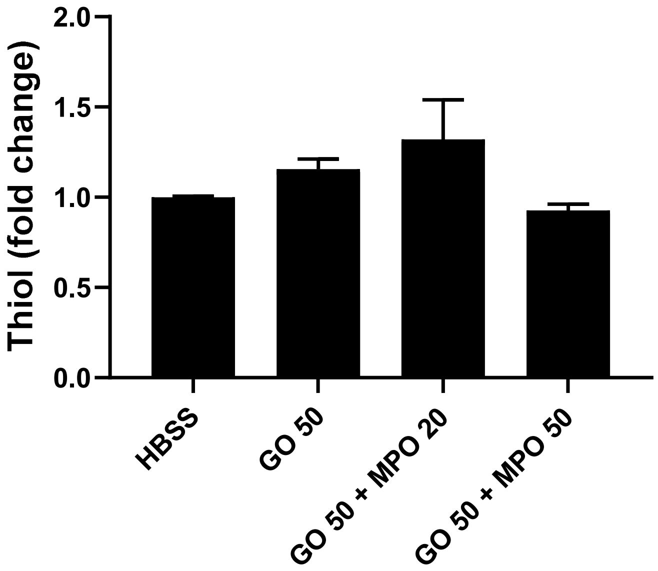

Figure S2: Effect of the glucose/GO/MPO enzymatic system on intracellular thiols in J774A.1 cells.

J774A.1 cells (5×10^5) were treated with GO (50 mU mL^{-1}) in the absence and presence of MPO (20 and 50 nM) in HBSS containing glucose (5.6 mM) for 1 h before re-incubation in cell media for 24 h. Intracellular thiols were quantified using ThioGlo 1 and normalised to the total protein concentration measured by BCA assay. Data are shown as the fold change of thiols compared to non-treated group and represent mean ± S.E.M from three independent experiments. Analysis by 1-way ANOVA with Dunnett's multiple comparison test showed no significant changes compared to the non-treated control (HBSS).

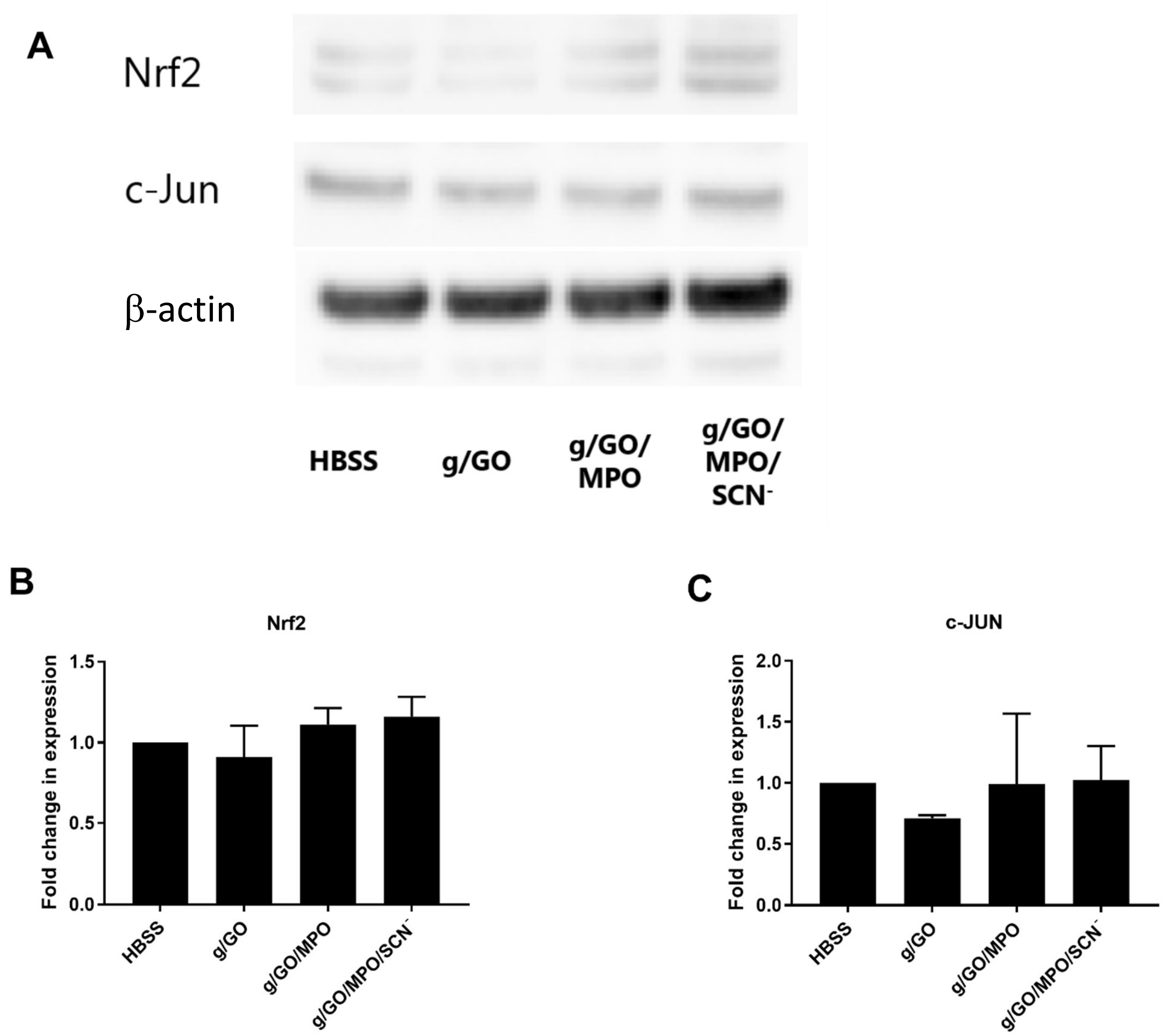

Figure S3. Cytoplasmic Nrf2 and c-JUN are not altered in J774A.1 cells on exposure to the glucose/GO/MPO enzymatic system.

J774A.1 cells (2×10^6) were incubated in HBSS containing glucose (5.6 mM) (black bars), with GO (50 mU mL^{-1}), GO/MPO (50 nM) or GO/MPO/SCN^- (200 µM) for 1 h at 37 °C before re-incubation in cell media for 24 h. The cytoplasmic protein was extracted using a commercial kit and 10 µg protein was loaded. β-actin was used as a loading control. Images are representative of 3 independent experiments (**A**). Panels **B** and **C** show the densitometry analysis of Nrf2 (**B**) and c-JUN (**C**) following normalization to β-actin. Data are expressed as the fold change compared to the respective non-treated group.
